# Antimicrobial Resistance in Members of the Bacterial Bovine Respiratory Disease Complex Isolated from Lung Tissue of Cattle Mortalities Managed with or without the Use of Antimicrobials

**DOI:** 10.3390/microorganisms8020288

**Published:** 2020-02-20

**Authors:** Kim Stanford, Rahat Zaheer, Cassidy Klima, Tim McAllister, Delores Peters, Yan D. Niu, Brenda Ralston

**Affiliations:** 1 Alberta Agriculture and Forestry, Lethbridge, AB T1J 4V6, Canada; 2 Agriculture and Agri-Food Canada, Lethbridge, AB T1J 4B1, Canada; rahat.zaheer@canada.ca (R.Z.); cassidyklima@gmail.com (C.K.); tim.mcallister@canada.ca (T.M.); 3 Alberta Agriculture and Forestry, Airdrie, AB T4A 0C3, Canada; delores.peters@gov.ab.ca; 4 Department of Ecosystem and Public Health, Faculty of Veterinary Medicine, University of Calgary, Calgary, AB T2N 1N4, Canada; dongyan.niu@ucalgary.ca

**Keywords:** *Mannheimia haemolytica*, *Pasteurella multocida*, *Histophilus somni*, *Mycoplasma bovis*, antimicrobial resistance, managed without antimicrobials, bovine respiratory disease

## Abstract

Over a two-year period, *Mannheimia haemolytica* (MH; *n* = 113), *Pasteurella multocida* (PM; *n* = 47), *Histophilus somni* (HS; *n* = 41) and *Mycoplasma bovis* (MB; *n* = 227) were isolated from bovine lung tissue at necropsy from cattle raised conventionally (CON, *n* = 29 feedlots) or without antimicrobials [natural (NAT), *n* = 2 feedlots]. Excluding MB, isolates were assayed by PCR to detect the presence of 13 antimicrobial resistance (AMR) genes and five core genes associated with integrative and conjugative elements (ICEs). Antimicrobial susceptibility phenotypes and minimum inhibitory concentrations (MICs, µg/mL) were determined for a subset of isolates (MH, *n* = 104; PM, *n* = 45; HS, *n* = 23; and MB, *n* = 61) using Sensititre analyses. A subset of isolates (*n* = 21) was also evaluated by whole-genome sequencing (WGS) based on variation in AMR phenotype. All five ICE core genes were detected in PM and HS by PCR, but only 3/5 were present in MH. Presence of *mco* and *tnpA* ICE core genes in MH was associated with higher MICs (*p* < 0.05) for all tetracyclines, and 2/3 of all macrolides, aminoglycosides and fluoroquinolones evaluated. In contrast, association of ICE core genes with MICs was largely restricted to macrolides for PM and to individual tetracyclines and macrolides for HS. For MH, the average number of AMR genes markedly increased (*p* < 0.05) in year 2 of the study due to the emergence of a strain that was PCR positive for all 13 PCR-tested AMR genes as well as two additional AMR genes (*aadA31* and *bla_ROB-1_*) detected by WGS. Conventional management of cattle increased (*p* < 0.05) MICs of tilmicosin and tulathromycin for MH; neomycin and spectinomycin for PM; and gamithromycin and tulathromycin for MB. The average number of PCR-detected AMR genes in PM was also increased (*p* < 0.05) in CON mortalities. This study demonstrates increased AMR especially to macrolides by bovine respiratory disease organisms in CON as compared to NAT feedlots and a rapid increase in AMR following dissemination of strain(s) carrying ICE-associated multidrug resistance.

## 1. Introduction

Bovine respiratory disease (BRD) is the leading cause of morbidity and mortality of North American feedlot cattle [1]. Multiple factors are potentially predisposing to BRD, including stress from weaning [2], transportation, co-mingling at auction markets [3], extreme weather changes [4], and changes in diet [5]. However, it is unclear whether these factors increase susceptibility and/or exposure to BRD pathogens or are confounded with poor management [4]. The bacterial pathogens, *Mannheimia haemolytica*, *Pasteurella multocida*, *Histophilus somni* and *Mycoplasma bovis* rapidly proliferate in the nasopharynx as a preliminary step in the development of BRD [5] and these organisms are also routinely detected in the lungs of BRD mortalities [6,7]. *M. haemolytica* has been the organism most frequently isolated from necropsied lungs of cattle with BRD in many studies [1,2,6,7], although age of cattle sampled, type of BRD, and antimicrobial therapies received also influence BRD organisms recovered [8,9,10].

Therapy at arrival to the feedlot (metaphylaxis) is a common industry practice to prevent BRD in high-risk cattle [11,12]. However, in many parts of the world, bacterial BRD pathogens are exhibiting a decrease in susceptibility to antimicrobials due to the development of antimicrobial resistance (AMR) [10,13,14,15]. Also of concern is the volume of antimicrobials used in prevention and/or treatment of BRD [16]. Although mechanisms of AMR are undoubtedly complex, exposure of bacteria to antimicrobials can select for resistant organisms [10] and with horizontal gene transfer, multidrug resistance cassettes can be shared across bacterial species or strains [2]. Accordingly, antimicrobial use in animal agriculture is under increased scrutiny due to its potential to increase AMR in human or veterinary pathogens.

In order to achieve effective global governance of AMR, a greater understanding of the complex relationships among AMR genotype and phenotype is required [14] as well as the role of antimicrobial use in development and spread of AMR [17]. The present study evaluated isolates of *Mannheimia haemolytica*, *Pasteurella multocida*, *Histophilus somni* and *Mycoplasma bovis* collected from necropsied lungs of feedlot cattle over a two-year period. Cattle were reared either conventionally or without the use of antimicrobials in an effort to increase understanding of relationships among antimicrobial use, AMR phenotype, and AMR genotype in bacteria associated with BRD. 

## 2. Materials and Methods 

### 2.1. Sample Collection

Four veterinary practices sampled BRD mortalities from 31 commercial feedlots in southern Alberta. Feedlots ranged in capacity from 5500 to 25,000 head and were either managed using conventional industry practices (CON, *n* = 29) or without the use of antimicrobials (NAT, *n* = 2). All available mortalities showing evidence of infectious pneumonia at post-mortem from either lung were sampled, (CON, *n* = 331; NAT, *n* = 19). Sections of lung tissue (3 cm^3^) from the perimeter of a visible lesion were collected in sterile containers and frozen at the veterinary practice (−18 °C) prior to weekly transport to the laboratory to enable collection of samples over a wide geographic area. At the laboratory, a sterile scalpel was used to excise a 1 cm^3^ section which was then homogenized in 10 mL brain heart infusion broth (BHI; BD Canada Inc., Mississauga, ON, Canada) in a stomacher (Seward model 400, Cole Palmer, Montreal, QC, Canada) at 230 rpm for 30 s. 

### 2.2. Isolation of Bacteria 

A 100 µL aliquot of lung tissue suspension was serially diluted in phosphate buffer solution (pH 7.4). 10^−1^ and 10^−2^ dilutions were plated (100 µL) onto tryptic soy agar with 5% sheep blood (BAP; Dalynn Biologicals, Calgary, AB, Canada) for the isolation of *H. somni* (HS) and blood agar modified with 15 µg/mL bacitracin (BAC; Dalynn) for the isolation of *M. haemolytica* (MH) and *P. multocida* (PM). BAC plates were incubated for 18–24 h at 37 °C and BAP plates for 48 h at 37 °C in 5% CO_2_. Three colonies exhibiting morphologies typical of MH, PM, or HS as described by Klima et al. [2] were sub-cultured onto fresh plates and incubated for 18–24 h at 37 °C (MH and PM) or 48 h at 37 °C in 5% CO_2_ (HS).

To isolate *M. bovis* (MB), 150 µL of the lung suspension was added to 1.5 mL of PPLO media (Thermo Fisher, Edmonton, AB, Canada) containing 500 µg/mL ampicillin before filtering through a syringe filter with a 0.2 µm sterile membrane (Acrodisc^®^, Pall Canada Ltd., Mississauga, ON, Canada). Filtrate ( 100 µL) was then plated on PPLO agar containing 500 µg/mL ampicillin (Dalynn) and incubated at 37 °C for 72–120 h in a 5% CO_2_ atmosphere Three colonies showing typical “fried egg” morphology [18] were sub-cultured onto a fresh PPLO plate and incubated for an additional 72–120 h at 37 °C in 5% CO_2_. For each of the BRD organisms, three sub-cultured colonies exhibiting typical morphology were stored separately in glycerol at −80 °C. Only one of the three colonies was further processed, with the others used as back-ups in case the first colony was not viable. An additional fourth suspect colony of each organism was stored in 1 mL of TE buffer (10 mM Tris, 1 mM EDTA, pH 8.0) at 4 °C until PCR analyses. 

### 2.3. PCR Confirmation of Species and Detection of AMR Genes

TE buffer stocks were heated to 95 °C for 5 min for heat lysis of DNA. A volume of 2 µL provided the DNA template for PCR confirmation of each species. All PCR runs included positive (ATCC3396, MH; ATCC37250, ATCC17976B, PM; ATCC70025, HS; ATCC25523, MB) and negative (nuclease-free water) controls. PCR were generated using HotStarTaq Plus master mix (Qiagen Canada Inc., Toronto, ON, Canada) and ran on a Verti™ Dx Thermal Cycler (Applied Biosystems, Burlington, ON, Canada). Primers and annealing conditions for MH, PM, and HS were as described by Klima et al. [2]. For MB, the three targets included *uvrC*, 16S rDNA, and *Mycoplasma* 16S to 23S rDNA intergenic transcribed spacer region with primers and annealing conditions as described by Gioia et al. [19]. Only PCR-confirmed MH, PM, HS, or MB isolates were included in subsequent analyses.

Heat-lysed DNA (2 µL per target) was also used for PCR detection of 13 AMR resistance genes (Table 1) that have been identified on an integrative and conjugative element (ICE) characterized in *Pasteurella multocida* [ICE*Pmu1*; 20]. An additional five core ICE-associated genes distributed across the length of ICE*Pmu1*, including hypothetical protein *02680-hyp*, integrase *2700-int1*, relaxase *2890-rel1*, multi-copper oxidase *mco*, and transposase *3510-tnpA* were also selected as PCR targets spanning the length of the ICE. Primers and PCR conditions for ICE-associated genes were as previously described [2,20,21,22] and were evaluated in isolates of MH, PM and HS.

### 2.4. Antimicrobial Susceptibility Phenotyping

A broth micro-dilution assay using a commercially available panel was used to evaluate antimicrobial susceptibility profiles of MH, PM and HS isolates (bovine/porcine with tulathromycin MIC format, Sensititre; Trek Diagnostic Systems, Cleveland OH, USA) according to manufacturer’s specifications. For MB, glycerol stocks were resuscitated in PPLO with 0.5% pyruvate and incubated at 37 °C in 5% CO_2_ for 72 h. A 150 µL subculture was then added to 15 mL PPLO with 0.5% pyruvate and incubated as described above for 48 h. From this culture, optical density (OD_450_) was measured using a Genesys 20 spectrophotometer (Thermo Scientific) to adjust cell concentration to approximately 10^8^ CFU/mL. A 60 µL aliquot of culture was then transferred to a 15 mL Falcon tube containing 6 mL PPLO broth without additives and mixed by inversion. Culture solution (50 µL) was then added to each well on a custom Sensititre panel which tested 10 antimicrobials over a range of dilutions (Table S1). Final cell concentration in wells was approximately 5 × 10^5^ CFU/mL. Wells were sealed and covered plates were incubated at 37 °C in 5% CO_2_ for 48 h. Positive and negative controls included MH ATCC3396 (MH); *Staphylococcus aureus* ATCC29213 (MH, PM, HS, MB); *Escherichia coli* O6 K-ATCC25922 (MH, PM, HS, MB); PM ATCC37250 (PM); PM ATCC17976B (PM), HS ATCC700025 (HS) and MB ATCC25523 (MB). The lowest concentration (µg/mL) of the antimicrobial inhibiting growth was defined as the MIC. When growth occurred at the highest antimicrobial concentration, the MIC was described as greater than the highest concentration. When no growth occurred at the lowest concentration, the MIC was expressed as less than the lowest concentration used. As MB is intrinsically resistant to β-lactams, all MB isolates exhibited growth at all concentrations of penicillin. 

### 2.5. Whole-Genome Sequencing and Analyses

Isolates for whole-genome sequencing (WGS) were selected from samples collected in year 1. Isolates were chosen based on a diversity of AMR phenotype and genotype determined by PCR and included those with consistently high or low MICs across antimicrobial classes. Bacterial isolates selected for whole-genome sequencing (WGS) were streaked from frozen glycerol stocks onto BAC plates for MH (*n* = 9) and PM (*n* = 5) and BAP plates for HS (*n* = 7). *Mycoplasma bovis* isolates were not included in WGS. Plates were incubated overnight at 37 °C for BAC and for 48 h at 37 °C in 5% CO_2_ for BAP. A single colony was sub-cultured with streaking onto BAC or BAP plates with appropriate incubation conditions for the bacterial species in question. Bacteria were grown in TE (10 mM Tris, 1 mM EDTA), pH 8.0 buffer until achieving an OD_600_ of~2, representing ~2 × 10^9^ cells/mL of suspension. The cell suspension (1 mL) was transferred to a microcentrifuge tube and centrifuged for 2 min at 14,000× *g*. Genomic DNA was extracted using DNeasy Blood and Tissue kit (Qiagen, Montreal, QC, Canada) following manufacturer’s instructions. DNA quality and quantity were estimated using a Nanodrop 2000 spectrophotometer and a Qubit Fluorometer with PicoGreen (Thermo Fisher Scientific, Mississauga, ON, Canada), respectively. Genomic library construction was performed using the Illumina Nextera XT DNA sample preparation kit (Illumina Inc., San Diego, CA, USA). Libraries were sequenced on an Illumina MiSeq platform using the MiSeq Reagent Kit V3 to generate 2 × 300 base paired-end reads.

Sequencing reads were assembled de novo into contigs using the SPAdes version 3.13.0 assembly pipeline that uses a multi-sized de Bruijn graph construction approach [23]. Draft genome assemblies were annotated with Prokka [24]. ABRicate version 0.8.7 (https://github.com/tseemann/ABRICATE) was used to search contigs against the NCBI Bacterial Antimicrobial Resistance Reference Gene Database (NCBI BioProject ID: PRJNA313047) to identify AMR genes and the presence of ICE in genomes.

### 2.6. Statistical Analyses

The overall prevalence of BRD bacteria isolated from mortalities was compared by generalized linear mixed models (Proc Glimmix, SAS 9.4, SAS Institute Inc, Cary, NC, USA) using a binomial distribution. Model-adjusted means (back transformed to the original scale) were reported, with mortality the experimental unit, veterinary practice a random effect, and *p* values <0.05 deemed significant.

Prior to any analyses of MICs, estimates were made for MICs either above or below concentrations of antimicrobials evaluated. For concentrations >256 µg/mL or <0.12 µg/mL, these were estimated as 256 and 0.12, respectively. Mid-range concentrations were set to the next highest or next lowest concentration commonly used on the Sensititre plate as appropriate. Original MIC values are shown in Supplementary Tables S1–S4. Distributions of estimated MICs were then evaluated using Proc Univariate. For combinations of organism and antimicrobials which followed approximately unimodal distributions, factors correlating with MICs were compared for each organism and antimicrobial combination using mixed model analyses. Bimodal distributions were evaluated using Proc Glimmix and a gamma distribution. For Glimmix analyses, model-adjusted means were back transformed to the original scale. For the organisms and antimicrobial combinations where MICs were fixed or for which gamma distributions were not appropriate, arithmetic means were calculated. In all analyses, type of management (CON, NAT), year and AMR/ICE-related gene fragments were fixed effects, with veterinary practice as a random effect. For MB as AMR and ICE core genes were not assayed, only type of management and year effects were evaluated. As no HS isolates were collected in NAT feedlots, and few HS isolates were collected in CON feedlots during year two, AMR and ICE core gene fragments were the only fixed effects for HS analyses. 

## 3. Results

### 3.1. Bacterial Isolation, AMR Phenotyping and PCR Detection of AMR/ICE-Core Genes

Recovery of MB from lung tissue was approximately twice that of MH (*p* < 0.001), with both MB and MH recovered more frequently than PM or HS (Table 2). Due to the abundance of MB, and limited availability of the custom plates, Sensititre analyses were restricted to a subsample of 61 isolates selected across collection dates, feedlots and management. Viability of HS stored within glycerol stocks was low (56%) in contrast to that of other members of the BRD bacterial complex (> 90%). This limited the number of HS isolates available for Sensititre analyses. 

The average number of AMR genes detected by PCR in MH isolates showed a 3-fold increase from year 1 to year 2 (*p* < 0.05, Table 3). Two MH isolates collected in year 1 from a single feedlot had 3/5 ICE core and 13/13 AMR genes, but by year 2 MH with the same suite of AMR and ICE core-related genes were identified in multiple feedlots within two veterinary practices and became the most commonly isolated strain of MH (39.6% of total; Table 4). Raising cattle with or without antimicrobials did not influence the total number of AMR genes detected by PCR in MH isolates. However, PM, isolates from CON feedlots had increased prevalence of AMR genes compared to NAT feedlots (*p* < 0.05) but the number of AMR genes present in PM isolates remained constant in isolates from both CON and NAT over both years of the study (Table 3). For PM, a strain with six AMR and the five ICE core genes was the most common in both years (Table 4). No isolates of HS were collected from NAT cattle, but diversity in carriage of AMR genes was higher in HS than MH, even though 2.6 x more MH isolates were collected. Lack of carriage of AMR genes was most common for HS (26.2% of isolates; Table 4) and 11/42 isolates had PCR AMR gene profiles which were different from those of all other isolates (data not shown). For MB, AMR and ICE core genes were not assayed by PCR as different primers would be required for sequence homology and work to develop these assays is still in progress. 

The number of ICE core gene fragments detected differed among organisms. For MH, *hypothetical protein* and *integrase* were not detected, while the prevalence of *transposase* and *mco* were identical (Table 5). For PM, prevalence of the five ICE core genes was identical in each isolate (Table 6). *Histophilus somni* was similar to PM in that the five ICE core genes were detected in some isolates, although carriage of individual ICE core genes varied among isolates. Isolates of HS having one, two, or three ICE core genes did not carry *mco*, but combinations of the other core genes varied (data not shown). For HS having four ICE core genes detected, *transposase* was absent (Table 7).

### 3.2. Effects of Management, Year of Study and AMR Genes on MICs 

Minimum inhibitory concentrations of antimicrobials for MB, MH, PM and HS prior to calculation of estimated MICs for statistical analyses are shown in Supplementary Tables S1–S4, respectively. For two antimicrobials (tulathromycin and tilmicosin), MICs for MH were higher (*p* < 0.05) for CON as compared to NAT-managed cattle (Table 5). Similarly, for PM, the MICs of two antimicrobials (neomycin and spectinomycin) were higher (*p* < 0.05) for CON as compared to NAT, although those for gentamicin were higher (*p* < 0.05) for NAT as compared to CON cattle (Table 6). Similar to MH, tulathromycin MICs were also higher (*p* < 0.05) for MB in CON as compared to NAT cattle (Table 8). 

Due to the limited number of viable HS isolates, it was not possible to determine the influence of year of study on HS MICs and for other organisms, year of study had mixed effects. For MH, MICs of clindamycin, chlortetracycline, and oxytetracycline were increased in year 2 (Table 5). For PM only, ampicillin MICs were increased in year 2 (Table 6), while for MB, the MICs for gamithromycin and tulathromycin decreased (*p* < 0.05) in year 2 (Table 8).

The presence/absence of AMR and ICE core genes was related to significant changes in MICs for some antimicrobials. Differences existed among the organisms evaluated, but in all cases where certain AMR and/or ICE core genes were detected and statistical analyses were performed, MICs were increased. For MH, presence of *mco*/*transposase* was associated with significant increase in MICs for six antimicrobials, while *relaxase* was only related to the MIC of clindamycin. 

Presence of ICE core genes in PM was associated with higher MICs (*p* < 0.05) for spectinomycin, tulathromycin tilmicosin and ceftiofur (Table 6). However, in contrast to MH the MICs of the majority of antimicrobials for PM either showed no variation or were not affected by the presence of AMR or ICE-related genes. Also noted for PM, isolates positive for *tet(H)* were also positive for *tet(R)* and the five ICE core gene fragments. In HS, MICs of only four antimicrobials were related to the presence of AMR or ICE core genes (Table 7). In contrast to PM, HS isolates positive for *tet(R)* were also positive for four ICE core genes, but not necessarily *tet(H)*. 

For some of the antimicrobials evaluated (enrofloxacin, danofloxacin, clindamycin, ceftiofur) no specific AMR-related genes were screened by PCR. For florefenicol, presence of *floR* had a greater influence on MIC than did presence of the ICE core genes in MH (Table 5) and HS (Table 7), as would be expected. Similarly, presence of *aph(3′)Ia* was the only influence on the MIC of neomycin in PM (Table 6), while *msr(E*) or *mph(E)* were the only influences on the MIC of tilmicosin in HS (Table 7). What was unexpected were the cases where ICE core genes predicted MICs better than did the presence of specific AMR-related genes. Examples of this later situation included chlortetracycline and oxytetracyline for MH (Table 5), tilmicosin for PM (Table 6) and tylosin for HS (Table 7). 

### 3.3. Sequence Analyses

The assembled draft genomes yielded average total genome sizes of 2.65 Mb for MH, 2.3 Mb for PM and 2.18 Mb for HS. Average numbers of contigs > 1 kb were 71, 35 and 35, respectively, for MH, PM and HS. Average N_50_ contig lengths for the three species were 91.3 kb, 163 kb and 130 kb, respectively. All sequenced MH belonged to serotype one as identified by the presence of a serotype 1-specific hypothetical gene (locus ID: D650_690 of USDA-ARS-USMARC-183; [25]). Out of nine MH sequenced from year 1, eight harbored AMR genes whereas no AMR genes were identified in one isolate (ID: MH9; Table 9). Six isolates carried the following five AMR genes within their ICE region: *aph(3″)-Ib* (previously known as *strA*), *aph(6)-Id* (previously known as *strB*), *aph(3′)-Ia* (previously known as *aphA-1*), *sul2*, and *tet(H)*, whereas two isolates had eight additional AMR genes in their ICE region, namely, *floR*, *erm(42)*, *ant(2″)Ia* (previously known as *aadB*), *aadA25*, *bla_OXA-2_*, *bla_ROB-1_*, *mph(E),* and *msr(E)* (Table 9). These two isolates carrying thirteen ICE-associated AMR genes also harbored nucleotide mutations producing amino acid changes in quinolone resistance-determining regions (QRDRs) of the *gyrA* (S83F and D87N) and *parC* (E89K) genes. Of five PM isolates sequenced, all but one harbored aminoglycoside resistance genes *aph(3″)-Ib*, *aph(6)-Id*, and *aph(3′)-Ia*, and all five carried *sul2*, *tet(H)*, and *aadA31* genes. Four AMR gene profiles were discovered in the 7 HS sequenced. The tetracycline resistance gene *tet(H)* was present in all 7 HS isolates. AMR genes *aph(6)-Id*, *aph(3″)-Ib*, and *sul2*,were present in other HS isolates. Four of six HS isolates also harbored *mph(E),* and *msr(E)*; one isolate carried *aph(3′)-Ia*, and *floR*, and one had *aph(3′)-Ia*, and *aadA31.* Along with the AMR genes, four HS also carried cation diffusion facilitator (CDF) family study to identify ICE using PCR are indicated. Two additional ICE-related genes *int2* and *parB* found to be conserved among ICE-containing isolates of MH, PM and HS are also marked. Gene *czcD* is known to facilitate the active efflux of one or more from a variety of metal cations including Zn, Cd, Ni, Co, Mn, and Cu, resulting in metal tolerance/resistance by efflux of ions [26].

**Figure 1:**
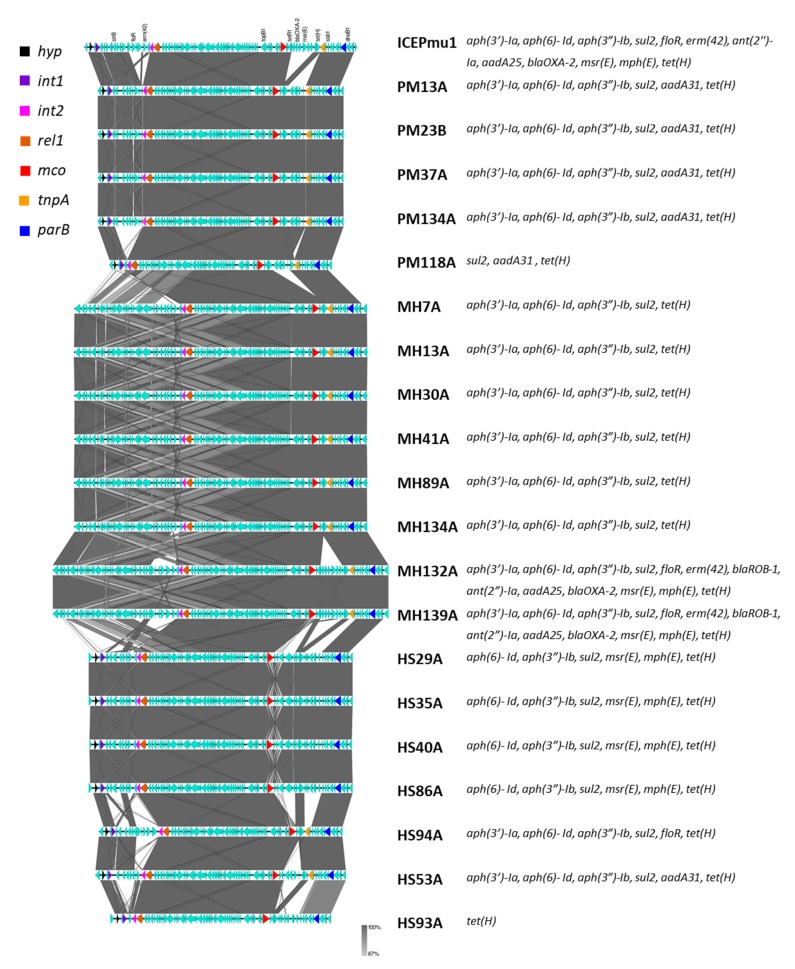
Schematics and homology of the ICE region in Pasteurellaceae species isolates determined by whole-genome sequencing [28]. Five ICE-related genes—*hyp*, *int1*, *rel1*, *mco* and *tnpA*—were employed in this study to identify ICE using PCR are indicated. Two addition ICE-related genes *int2* and *parB* found to be conserved among the ICE-containing isolates of MH, PM and HS are also marked. Most conserved genes present in the ICE region in sequenced isolates had 100% nucleotide sequence identity across three species.

Whole-genome sequence data indicated that in all ICE-harboring isolates of investigated Pasteurellaceae species ICE sequences were integrated within a chromosomal tRNA^Leu^ gene. Of the five ICE core genes (*hyp*, *int1*, *rel1*, *mco*, *tnpA*) associated with ICE*Pmu1* and investigated in this study for their presence with PCR, all five genes were present in all five PM sequenced here; all five genes were present in two of seven of the HS, while four genes but *tnpA* were present in the remaining five HS isolates sequenced (Figure 1). In all sequenced MH, only three (*rel1*, *mco*, *tnpA*) of five ICE core genes investigated were present. Sequence data indicated that an integrase-like gene with only 46% amino acid identity with *int1* encoding integrase protein and no notable homology at the nucleotide level was present in MH at a location similar to *int1* in PM and HS (Figure 1) but coded by the opposite strand.

In Pasteurellaceae species, along with many other genes related to ICE replication, conjugative transfer and chromosomal integration, three genes (also present in ICE*Pmu1*) *int2* (Pmu02880 in ICE*Pmu1*), a paralogue of *int1* XerD-family tyrosine integrase Pmu02700 [27], *rel1*, the ICE-*relaxase* (Pmu02890 in ICE*Pmu1*), and *parB* (Pmu03590 in ICE*Pmu1*), encoding partitioning protein associated with ICE replication [27] were among the most conserved genes present in the ICE region in sequenced isolates with 100% nucleotide sequence identity across three species.

## 4. Discussion

### 4.1. Recovery of Isolates

Factors contributing to BRD are complex and may include a variety of stressors such as viral infections [10] in addition to the stressors routinely associated with weaning, transport and feedlot entry [29,30,31]. Both stress and exposure to BRD pathogens disrupt the respiratory tract microbiome and result in BRD [32]. However, as BRD organisms are also found in healthy cattle [33] and sampling BRD cases has detected multiple respiratory pathogens [2,7,33], it is difficult to determine the organism(s) responsible for the mortality. 

In a related study which used a subset of the mortalities evaluated in the present study (*n* = 18) and evaluated bronchial lavage, MH was the primary BRD pathogen isolated [34]. Type of pneumonia responsible for the mortality [6] likely affected the BRD pathogens isolated in the two related studies. Sampling only lung tissue in the current study may have also increased isolation of MB as this bacterium is less-frequently found in the upper respiratory tract [5]. Number of antimicrobial treatments, classes of drugs received prior to death and drug kinetics also affect the respiratory microflora detected [33], but these data were not provided for CON managed cattle for reasons of confidentiality. For NAT feedlots, cattle showing symptoms of BRD were administered antimicrobials, moved to CON management at a separate location, and not sampled. NAT mortalities sampled did not receive antimicrobial therapy due to either a sudden onset or minimal symptoms of BRD. This situation further complicated comparison of CON and NAT management, in addition to the disparity among the numbers of feedlots sampled per management type. However, animal welfare considerations would preclude evaluation of NAT cattle which were not provided antimicrobial therapy after presumptive diagnosis of BRD.

In the present study, MH was the second-most common BRD organism isolated, with similar and lower numbers of PM and HS collected. Age of cattle and time on feed also affect BRD organisms detected. In young recently-weaned calves, PM has been the most common BRD pathogen identified [5]. Only 56% of HS isolates could be re-cultured for Sensititre analyses and difficulty in culturing HS [35] may lower the frequency of isolation of this organism. Freezing lung tissue prior to isolation may have also contributed to a low recovery of HS. However, freezing facilitated sample collection from veterinary practices located up to 250 km from the laboratory. 

### 4.2. Year and Management Effects on PCR Detection of AMR and ICE Genes

The AMR and ICE core gene fragments detected by PCR have been previously reported in MH, PM and HS [2,36], but not in MB [37]. As our laboratory did not have the necessary assays developed, MB isolates were not screened for AMR and ICE core genes. ICE*Pmu1* was initially identified in PM [20] but components of this ICE have also been identified in MH [36], and other closely-related ICE sharing 99% homology in conserved regions are carried by MH and HS [38]. However, ICEs carried by PM and HS share highest identities compared to those of MH [36]. In MH, two ICE core genes originating from ICE*Pmu1* investigated in this study, namely *hyp* and *int1* were not detected by PCR. Whole-genome sequence data confirmed that *hyp* gene was not present in MH whereas an orthologue of PM/HS *int1* with no notable sequence homology at the nucleotide level and very low (46%) amino acid sequence identity was present in MH. Analyses of the whole-genome sequences of three Pasteurellaceae species investigated here reveal that five genes—*int2*, *rel1*, *mco*, *parB* and *tetH*—appear to be always present in the ICE region and are conserved among these species with 100% nucleotide identity and, therefore, may be suitable candidates for PCR-based identification of ICE across all of these species. Recently, Beker et al. [27] developed a multiplex PCR assay targeting four ICE core genes *par*B, ICE-*rel1*, *int1* and *int2* related to integration and maintenance of ICE structures. Their results also indicate that *int1* was present only in a subset of ICEs. 

Due to the absence of a cell wall and limited numbers of metabolic pathways, only a few antimicrobials (tetracyclines, macrolides and some fluoroquinolones) are able to control MB infections [39] and AMR genes assayed in the present study affecting β-lactam, aminoglycoside or sulfonamide antimicrobials may only indirectly affect AMR in MB. Recently, genetic mutations reducing susceptibility of MB to fluoroquinolones, tetracyclines, macrolides, lincosamides, phenicols and pleuotulins have been reported [40], although PCR assays to detect these sequences were not in place at the time of this study. The genome of MB contains integrative and conjugative elements, but horizontal gene transfer by these elements in MB has yet to be demonstrated [41]. For MB, the WGS required prior to development of PCR primers for ICE and AMR genes is still in progress by our laboratory. Accordingly, analyses of year and management effects on AMR and ICE core genes in the present study evaluated only isolates of MH and PM. 

Antimicrobial resistance occurrence in BRD organisms of North American cattle has been gradually increasing over time especially to macrolides [10,14,15]. However, the marked jump in AMR-related genes and detected in MH isolates in year two of the current study is of concern and can be attributed to increased prevalence of a strain carrying 13 AMR and 3/5 ICE core genes (Table 3). This MH strain was first identified in a single CON feedlot in year one but spread to multiple CON feedlots in year 2. Due to interactions among multiple organisms, health status of host, virulence of pathogen(s), stage of disease when therapy starts, and susceptibility of organism(s), efficacy of antimicrobials for control of this strain of MH in vivo would be difficult to predict [10]. 

In contrast to MH, AMR genes did not increase over time in PM, although use of antimicrobials increased (*p* < 0.05) AMR genes detected. The strain of PM carrying six AMR and five ICE genes (Table 4) was not detected in any NAT feedlots, but calves in NAT feedlots would likely be directly sourced from a few trusted suppliers instead of co-mingled at auction as high-risk calves would be avoided. Consequently, the differences in AMR genes present in PM may be related to restricted calf origin and less to exposure to BRD organisms from multiple sources, as the respiratory tract resistome composition has significantly differed by geographical origin of cattle and has not always been influenced by prior use of antimicrobials [42]. 

Other studies of calves raised conventionally or without antimicrobials concluded that use of antimicrobials did not increase AMR of fecal *Escherichia coli* [43], organisms isolated from beef [44], or genes associated with carbapenem resistance in feces [45]. Although Vikram et al. [46] found increased detection of some macrolide and tetracycline-related AMR genes in feces of CON as compared to NAT calves, these differences were considered of minor importance. Although only two NAT feedlots were sampled, raising calves without antimicrobials significantly reduced the AMR genes present in PM. Conflicting results between this and earlier studies may be due to the bacterial species evaluated. As metaphylaxis primarily targets BRD bacterial pathogens, the selection pressure for AMR genes in BRD organisms under CON management may be higher than that in the commensal fecal microflora evaluated in previous studies. 

### 4.3. Concordance of AMR Phenotype and Genotype

In addition to PCR identification of AMR genes, we further investigated a small number of isolates of MH, PM and HS by WGS using Illumina platform and evaluated the genetic basis and nature of AMR phenotypes. All isolates carried ICE except for one MH (data not shown). In the present study, tetracycline resistance gene *tet(H)* as well as up to two copies of its repressor gene *tet(R)* were always present in isolates with ICE. Previous studies have also indicated the presence of ICE associated with *tet(H)* in MH, PM and HS [38,47,48]. In MH and PM, complete linkage of *aph(3″)-Ib*, *aph(6)-Id*, *aph(3′)-Ia*, and *sul2* was found in both WGS and PCR datasets. Other studies have also reported similar linkage of these aminoglycoside and sulfonamide/sulfamethoxazole resistance genes in MH [46]. In contrast, for HS *aph(6)-Id*, *aph(3′)-Ia*, and *sul2* co-occurred with *aph(3″)-Ib* either present or absent. A lack of concordance for *aph(6)-Id* detection between PCR and WGS occurred in 4/7 HS due to low nucleotide sequence identity (61%) between the *aph(6)-Id*/*strB* gene from ICE*Pmu1* targeted for PCR and its homolog in HS and, therefore, a lack of primer annealing. However, for 2/7 HS, *aph(6)-Id* was detected by PCR, indicating multiple alleles within HS for this gene. In MH and PM, spectinomycin resistance appeared to be caused by *aadA25*, or a newly discovered gene *aadA31* [49]. However, spectinomycin resistance phenotype in HS could not be explained. This resistance has also been found linked with 16S rRNA and mutations [50]. 

Aminoglycoside resistance mechanisms include enzymatic inactivation of the antibiotic molecule, modification of the target by mutation of the 16S rRNA or ribosomal proteins, reduced outer membrane permeability, inner membrane transport, active efflux pumping of drug molecules, and drug sequestration via tight binding to a low activity acetyltransferase [51]. Aminoglycoside resistance occurs through one or many of these mechanisms that can coexist in the same cell. The macrolide resistance gene phenotype appeared to be more co-related to the presence of *erm(42)*, and macrolide efflux protein and phosphotranferase gene pair *msr(E)*-*mph(E)* in HS than in MH or PM. Macrolide resistance due to rRNA mutations is already well documented in bacteria with a single (or a few) rrn operons [52]. The 23rRNA gene in assembled sequences of MH and PM appeared to be wild-type versions. However, it is possible that a mutation is present in a few but not all copies of 23S rRNA. This may have resulted in the macrolide resistant phenotype or that of another uncharacterized rRNA methyltransferase in these isolates. Two MH isolates and one PM isolate exhibiting phenotypic resistance to fluoroquinolones had relevant mutations (Table 9) in quinolone resistance-determining regions (QRDRs) as previously reported [53,54], in DNA-gyrase encoded by the *gyrA* and *gyrB* genes and Topoisomerase IV encoded by *parC* and *parE* [55]. Fluoroquinolone resistance can occur through mutations in genes coding for the antimicrobial target enzymes (DNA gyrase and topoisomerase IV) but can also be plasmid mediated [56]. In Enterobacteriaceae, qnrS, qnrB and aac(6′)-Ib-cr genes have been implicated in plasmid-mediated quinolone resistance (PMQR) [57,58]. A combination of low-level resistance to fluoroquinolones caused by PMQR mechanism, with QRDR mutation can lead to clinically relevant resistance [59]. We did not find any PMQR in the fluoroquinolone resistant isolates exhibiting QRDR, indicating that these mutations were only identified in resistant isolates. 

### 4.4. Factors Affecting MICs

Influences of year of study on MICs were minimal and unlikely to be due to changed antimicrobial use in CON feedlots, as the number of antimicrobials used remained similar in both years (data not shown). As multiple factors would affect MICs, two years is likely too short of a period for consistent trends to emerge. Gautier-Bouchardon et al. [60] analyzed MICs for MB from cattle in France over 30 years, finding substantial increases for some macrolides (tylosin, tilmicosin, tulathromycin) and not others (gamithromycin, tildipirosin. In North America, CON calves would usually get a metaphylactic injection of tulathromycin on arrival at the feedlot [61] and tulathromyin is also used to treat active cases of BRD. Exposure to tulathromycin has increased its MIC in MH and not PM [62], in agreement with the results of our study for MH, although increased MICs for tulathromycin could also be influenced by co-selection. The present study also found CON management increased the MIC of tulathromycin for MB, which is of concern due to current difficulty controlling MB infections [63] without considering future increases in AMR.

For two antimicrobials (sulfadimethoxine, clindamycin), PM MICs were the same regardless of type of management, presence of ICE core or AMR gene fragments, or year of study. As clindamycin is most often used in companion animals and has never been registered for use in Canadian cattle [64], fixed clindamycin resistance is not likely due to previous exposure of cattle to clindamycin. *Erm(42)* has been reported to markedly increase MICs for clindamycin [65], but this gene was not detected in PM. Accordingly, other uncharacterized mechanisms for clindamycin resistance/co-resistance were likely responsible for the fixed clindamycin resistance observed in PM.

Vikram et al. [45] found increased tetracycline resistance in feces of CON as compared to NAT calves, but type of management in the present study did not affect MICs of oxy- or chlortetracycline for MH, PM or MB. For PM, if *tet*(H) was present, *tet*(R) was also present as were the 5 ICE core genes. In contrast, for MH *tet*(H) and *tet*(R) were present at different frequencies compared to ICE core genes, Sequence analyses of MH in a companion study [34] revealed diverse AMR gene carriage by ICE. Deletions have been found in AMR genes carried by ICE in MH [36] and may have contributed to variable carriage of *tet*(H) and *tet*(R) in relation to MH ICE core genes in the present study. In all isolates sequenced, apart from multiple profiles (presence/absence) of AMR genes, the synteny of ICE-associated genes was similar to ICE*Pmu1*, ICE*Mh1* or ICEMhL044A [20,25,66], likely due to horizontal transfer of ICE among these members of the BRD bacterial complex.

For HS, presence of ICE core genes affected relatively few MICs compared to the presence of these genes in either PM or MH, but few statistical analyses were performed as many HS distributions were fixed or if bimodal could not be modelled using a gamma distribution. ICE*Hs1* which has been isolated from HS in Alberta feedlot cattle has been shown to carry *tet(H)* and *mco* but not *tet(R)* [38]. In contrast, results of the present study showed *tet(R)* was present at the same frequency as core ICE genes and WGS determined that *tet(R)* was located within HS ICE. AMR genes were diverse for all BRD pathogens and AMR genes other than those assayed could be carried on ICE and affect MICs. For example, tylosin HS MICs were related only to the presence of the four ICE gene fragments with identical prevalence and not to any of the macrolide-specific AMR genes assayed, implying that some other ICE constituent is contributing to AMR observed. Similarly, for oxytetracycline and chlortetracycline, MH MICs were related to presence of *mco*/transposase and not to *tet(R*) or *tet(H),* while PM MICs for macrolides were related to core ICE genes and not to *erm*(42), *mph*(*E)* or *msr(E).*

For MB, raising cattle with or without antimicrobials affected MICs of the macrolides gamithromycin and tulathromycin, which were higher in CON mortalities. Regardless of management, MICs of florfenicol and oxytetracycline were lower for MB in the current study than those reported in France from 2010–2012 [60], although MICs for macrolides were higher with the exception of that for tilmicosin in Alberta as compared to French cattle. Maunsell et al. [63] compiled studies of MB antimicrobial susceptibility reporting MICs for tilmicosin and florfenicol similar to those of the current study, while those of oxytetracycline and tetracycline had considerable location-specific variation. As MICs reported for BRD organisms show geographical differences, local studies are needed to better characterize existing AMR and changes over time.

## 5. Conclusions

This study demonstrated a rapid increase in AMR, following dissemination of MH strain(s) carrying ICE-associated multi-drug resistance. It is unlikely that presence of ICE core genes directly increased MICs. Instead, ICE core genes served as markers for the presence and possible interactions among the suite of AMR genes present in different regions of the ICE. For MH, presence of these gene fragments was associated with increased MICs for all tetracyclines, and 2/3 of the macrolides, aminoglycosides and fluoroquinolones evaluated. For both PM and HS, presence of ICE core genes had less relation to MICs, and these relationships were largely restricted to macrolides for PM and to individual tetracyclines and macrolides with HS. All five ICE core genes investigated here via PCR were detected in PM and HS, but only 3/5 could be detected in MH due to the lack of conservation of two of the genes (*hyp* and *int1*) across species. PCR and WGS data collectively identified five ICE-associated genes—*int2*, *rel1*, *mco*, *parB* and *tetH*—that were distributed along the length of the ICE structures and were highly conserved across PM, MH and HS. These genes could, therefore, be suitable PCR targets for ICE detection in these Pasteurellaceae species BRD pathogens. Based on results of the present study, BRD mortalities which were NAT managed generally had reduced MICs for MH, PM and MB across classes of antimicrobials and for PM, significantly fewer AMR-related gene determinants were detected in isolates collected from NAT mortalities. However, management of cattle without antimicrobials is not without cost and it is not presently possible to predict whether the differences in MICs noted would result in reduced efficacies of antimicrobial therapies in CON as compared to NAT cattle. Of all AMR evaluated, the increased MIC for tulathromycin in CON mortalities for both MH and MB is of special concern due to the reliance on this antimicrobial for BRD metaphylaxis. Future studies evaluating BRD will verify the extent to which changes in feedlot management practices can contribute to retaining the efficacy of existing antimicrobials.

## Figures and Tables

**Table 1:** Antimicrobial resistance and ICEP*mu1* core gene targets for PCR-based detection.

Phenotype/Function	PCR Target(s)	Base Pairs	T ^z^	Reference
Antimicrobial resistance class				
Aminoglycoside				
gentamicin	*ant(2″)-Ia*	551	66	[2]
neomycin	*aph(3′)-Ia*	489	54	[21]
spectinomycin	*aadA25*	503	66	[2]
streptomycin	*aph(3″)-Ib*	506	64	[2]
	*aph(6)-Id*	586	64	[2]
Amphenicol	*floR*	320	58	[2]
Macrolide	*msr(E) mph(E) erm(42)*	620,401, 1254	60	[2]
Penicillin	*bla_OXA_*_-2_	685	60	[22]
Sulfonamide	*sul*2	489	64	[2]
Tetracycline	*tet*(*H*) *tet(R)*	1076	60	[22]
Ice core genes				
Hypothetical protein	*Pmu*02680	226	58	[2]
Integrase (*int1*)	*Pmu*02700	735	58	[2]
Multi-copper oxidase (*mco*)	*Pmu*03360	458	58	[2]
Transposase (*tnpA*)	*Pmu*03510	204	56	[2]
ICE-Relaxase (*rel*)	*Pmu*02890	695	57	[20]

^z^ T, Annealing temperature, °C.

**Table 2:** Overall prevalence of isolates recovered from lung tissue of bovine respiratory disease mortalities (*n* = 350).

Organism	# of Isolates Collected	Overall Prevalence (%)	# of Isolates with Sensititre Analyses ^z^	# NAT Isolates with Sensititre Analyses
***Mannheimia haemolytica***	113	31.6 ± 2.2 ^b^	104	6
***Pasteurella multocida***	47	12.9 ± 2.2 ^a^	45	4
***Histophilus somni***	41	12.5 ± 1.4 ^a^	23 ^y^	0
***Mycoplasma bovis***	227	63.9 ± 3.8 ^c^	61 ^x^	7

^a,b,c^ Means in a column with different superscripts differ (*p* < 0.001); ^z^ Only one isolate of each organism per lung tissue sample was evaluated with additional isolates evaluated only in case of poor viability; ^y^ Isolates of *H. somni* for 18 tissue samples were not viable; ^x^ A subset of *M. bovis* isolates was selected for analysis due to limited availability of custom plates.

**Table 3:** Effect of year and feedlot management on numbers of AMR-related genes detected by PCR in isolates of *Mannheimia haemolytica* and *Pasteurella multocida* from lung tissue of bovine respiratory disease mortalities.

Organism	Management ^z^		Year of Study		SEM
	CON	NAT	1	2	
*Mannheimia haemolytica*	6.7	4.1	2.6 ^a^	8.2 ^b^	2.8
*Pasteurella multocida*	4.4 ^b^	0.5 ^a^	3.9	3.8	2.3
*Histophilus somni*	2.8	NA ^y^	2.8	NA	NA

^a,b^ Means within a row with different superscripts differ (*p* < 0.05); ^z^ Management, CON = conventional, NAT =without antimicrobials; ^y^ NA, not applicable due to limited isolates collected.

**Table 4:** The three most common AMR gene PCR patterns ^z^ for *M. haemolytica* (MH), *P. multocida* (PM) and *H. somni* (HS) isolated from lung tissue of bovine respiratory disease mortalities. ‘+’ = present, ‘-’ = absent; ICE core gene fragments^y^; AMR gene fragments.

Strain	*hyp*	*int*	*rlx*	*mco*	*tnp*	*aph(3′) Ia*	*aph(6)Id*	*aph(3″)Ib*	*sul2*	*floR*	*erm (42*)	*ant(2″)Ia*	*aadA25*	*bla _OXA-2_*	*msr (E)*	*mph (E)*	*tet (R)*	*tet (H)*	Total Genes	# Isolates with Same Pattern (% of Total Isolates)
MH	-	-	+	+	+	+	+	+	+	+	+	+	+	+	+	+	+	+	16	44/111 (39.6)
MH	-	-	+	-	-	-	-	-	-	-	-	-	-	-	-	-	-	-	1	23/111 (20.7)
MH	-	-	+	+	+	+	+	+	+	-	-	-	-	-	-	-	+	-	8	14/111 (12.6)
PM	+	+	+	+	+	+	+	+	+	-	-	-	-	-	-	-	+	+	11	22/47 (46.8)
PM	-	-	-	-	-	-	-	-	-	-	-	-	-	-	-	-	-	-	0	10/47(21.3)
PM	+	+	+	+	+	-	-	-	-	-	-	-	-	-	-	-	+	+	7	9/47(19.1)
HS	-	-	-	-	-	-	-	-	-	-	-	-	-	-	-	-	-	-	0	11/42(26.2)
HS	+	+	+	+	-	-	-	-	+	+	-	-	-	-	+	+	+	+	10	7/42 (16.7)
HS	+	+	+	+	-	+	+	+	+	+	-	-	-	-	+	+	+	+	13	3/42 (7.1)

^z^ Additional information regarding PCR primers shown in Table 1. ^y^ ICE core gene fragments: *hyp*, hypothetical protein; *int*, integrase; *rlx*, relaxase; *mco*, multi-copper oxidase; *tnp*, transposase.

**Table 5:** Effect of management ^z^, year of study and presence of ICE core and antimicrobial resistance genes of minimum inhibitory concentrations (MICs) for *M. haemolytica* (*n* = 104). Least square means are reported for antimicrobials showing unimodal (unshaded) or selected bimodal distributions (shaded in light grey). Arithmetic means are reported for other distributions (shaded in dark grey). MICs < or > than the range of concentrations evaluated were estimated as the next lower or higher concentration commonly used on the Sensititre plate.

Antimicrobial	CON	NAT	1	2	SEM	Gene	+	-	Gene	+	-	Gene	+	-
Ampicillin	15.4	14.3	14.1	15.7	NA ^y^	*bla_OX-A2_*	29.5	0.2	*relaxase*	14.8	14.9	*mco*	15.2	14.1
Ceftiofur	0.2	0.0	0.4	0.0	0.9	*relaxase*	0.6	0.0	*mco^x^*	0.0	0.2			
Clindamycin	20.3	16.2	16.5 ^a^	19.9 ^b^	2.3	*relaxase*	27.3 ^b^	9.2 ^a^	*mco*	18.2	18.2			
Chlortetracycline	2.1	1.9	1.5 ^a^	2.7 ^b^	0.6	*tet(H)*	2.2	1.9	*tet(R)*	1.7	2.4	*relaxase*	2.5	1.6
						*mco*	4.4 ^b^	0.9 ^a^						
Danofloxacin	0.6	0.3	0.1	0.8	NA	*relaxase*	0.4	0.5	*mco*	0.9	0.0			
Enrofloxacin	1.2	0.7	0.2	1.6	NA	*relaxase*	1.1	0.7	*mco*	1.9	0.0			
Florfenicol	3.7	3.8	4.1	3.4	0.6	*floR*	14.6 ^b^	0.9 ^a^	*relaxase*	4.4	3.1	*mco*	3.5	4.0
Gentamicin	11.2	11.8	11.6	11.4	2.2	*ant(2″)-Ia*	43.7 ^b^	3.0 ^a^	*relaxase*	15.1 ^b^	8.8 ^a^	*mco*	11.2	11.9
Neomycin	23.0	22.3	22.6	22.7	0.8	*aph(3″)Ia*	25.2 ^b^	20.3 ^a^	*relaxase*	22.7	22.6	*mco*	63.3 ^b^	8.1 ^a^
Oxytetracycline	3.5	3.3	1.7 ^a^	4.2 ^b^	0.9	*tet(H)*	4.1	2.8	*tet(R)*	3.0	3.8	*relaxase*	4.4	2.6
						*mco*	10.2 ^b^	1.1 ^a^						
Penicillin	7.5	7.0	6.9	7.7	NA	*bla_OX-A2_*	13.4	1.2	*relaxase*	7.2	7.3	*mco*	7.5	7.0
Spectinomycin	77.6	50.7	63.1	65.3	11.4	*aadA25*	98.0 ^b^	30.3 ^a^	*relaxase*	61.2	67.1	*mco*	72.9 ^b^	55.4 ^a^
Sullfadimethoxine	254.6	206.4	228.8	232.2	NA	*sul*2	227.9	233.0	relaxase	228.6	232.5	*mco*	233.1	228.0
Tilmicosin	63.2 ^b^	30.2 ^a^	48.2	45.2	12.5	*msr(E)*	89.6 ^b^	3.8 ^a^	*mphE*	55.7	37.7	*erm(42)*	76.5 ^b^	17.0 ^a^
						*relaxase*	52.2	41.2	*mco*	84.3 ^b^	9.1 ^a^			
Trimethroprim/ Sulfmethazole	0.05	0.02	0.04	0.03	NA	*sul*2	0.02	0.05	relaxase	0.07	0.00	*mco*	0.06	0.02
Tulathromycin	61.1 ^b^	28.9 ^a^	45.8	28.9	12.8	*msr(E)*	86.1 ^b^	3.9 ^a^	*mph(E)*	53.1	36.9	*erm(42)*	72.7 ^b^	17.4 ^a^
						*relaxase*	54.8	35.2	*mco*	54.8 ^b^	9.3 ^a^			
Tylosin	63.0	67.0	60.9	69.1	NA	*msr(E)*	66.2	63.8	*mph(E)*	63.2	66.2	*erm(42)*	55.1	65.2
						*relaxase*	62.8	67.2	*mco*	68.4	62.8			

^a,b^ Means within a row with different superscripts differ (*p* < 0.05). ^z^ Management, CON = conventional, NAT= without antimicrobials; ^y^ NA, not applicable as arithmetic mean. ^x^ Transposase TNP3510 and *mco* present at identical frequencies.

**Table 6:** Effect of management ^z^, year of study and presence of ICE core and antimicrobial resistance genes on of minimum inhibitory concentrations (MICs) for *P. multocida* (*n* = 45). Least square means are reported for antimicrobials showing unimodal distributions (unshaded) and arithmetic means for other distributions (shaded in dark grey). MICs < or > than the range of concentrations evaluated were estimated as the next lower or higher concentration commonly used on the Sensititre plate.

Antimicrobial	CON	NAT	1	2	SEM	Gene	+	-	Gene	+	-	Gene	+	-
Ampicillin	11.7	21.3	10.7 ^a^	22.3 ^b^	6.1	*bla_OXA-2_*	NA^v^		ICE	20.0	13.0			
Ceftiofur	6.1	11.1	7.7	9.4	3.2	ICE^y^	11.0 ^b^	6.1 ^a^						
Chlortetracycline	2.1	0.4	1.3	0.7	1.5	*tet(H), tet(R)*	0.8	1.1	*ICE*	NA^x^				
Clindamycin	32.0	32.0	32.0	32.0	NA^w^	ICE	32.0	32.0						
Danofloxacin	0.33	0.20	0.24	0.29	0.29	ICE	0.44	0.09						
Enrofloxacin	0.13	0.06	0.12	0.07	0.39	ICE	0.17	0.02						
Florfenicol	1.0	1.2	1.4	0.8	1.2	*floR*	NA^u^		*ICE*	1.4	0.9			
Gentamicin	5.4 ^a^	17.1 ^b^	10.7	11.9	3.3	*ant(2″)-Ia*	NA^w^		*ICE*	11.8	10.8			
Neomycin	43.2 ^b^	20.9 ^a^	34.3	29.1	6.1	*aph(3′)Ia*	55.9 ^a^	8.1 ^b^	*ICE*	27.3	36.8			
Oxytetracycline	8.1	7.6	9.1	6.5	3.7	*tet(H)*, *tet(R)*	10.2	5.4	ICE	NA^x^				
Penicillin	5.3	7.7	4.9	8.1	3.8	*bla_OXA-2_*	NA^v^		ICE	8.3	4.7			
Spectinomycin	94.0 ^a^	37.8 ^b^	69.0	57.9	11.2	*aadA25*	NA^u^		ICE	93.3 ^b^	33.5 ^a^			
Sulfadimethoxine	256	256	256	256	NA^w^	*sul*2	256	256	ICE	256	256			
Tilmicosin	66.6	54.4	70.4	50.6	25.8	*msr(E) mph(E)*	NA^u^		*erm(42)*	NA^u^		ICE	94.2 ^b^	26.8 ^a^
Trimethroprim/ Sulfmethazole	0.08	0.09	0.09	0.08	NA^w^	*sul*2	0.09	0.08	ICE	0.10	0.07			
Tulathromycin	63.3	61.5	72.0	52.8	26.1	*msr(E) mph(E)*	NA^u^		*erm(42)*	NA^u^		ICE	99.6 ^b^	25.2 ^a^
Tylosin	48.3	41.1	48.2	41.2	NA^w^	*msr(E) mph(E)*	NA^u^		*erm(42)*	NA^u^		ICE	57.2	32.1

^a,b^ Means within a row with different superscripts differ (*p* < 0.05). ^z^ Management, CON = conventional, NAT = without antimicrobials; ^y^ ICE, all five ICE core gene fragments present in equal frequencies. N/A^x^, isolates positive for *tet(H)* positive for *tet(R)* and five ICE core gene fragments. NA^w^, not applicable as arithmetic mean; NA^v^, no isolates positive for this gene fragment. NA^u^, only one isolate positive for this gene(s).

**Table 7:** Effect of presence of ICE core and antimicrobial resistance genes on minimum inhibitory concentrations (MICs) for *H. somni* (*n* = 23). Least square means are reported for antimicrobials showing unimodal distributions (unshaded). Arithmetic means are shaded in dark grey. MICs < or > than the range of concentrations evaluated were estimated as the next lower or higher concentration commonly used on the Sensititre plate.

Antimicrobial	Gene	+	-	Gene	+	-	Gene	+	-	Gene	+	-
Ampicillin	*bla_OXA-2_*	NA^x^		ICE^z^	4.0	0.0	*TNP*	0.0	3.4			
Ceftiofur	*ICE*	0.08	0.03	*TNP*	0.11	0.00						
Chlortetracycline	*tet(H)*	1.8	0.9	*tet(R)*/ICE^y^	3.0 ^b^	0.0 ^a^	*TNP*	2.3 ^b^	0.3 ^a^			
Clindamycin	*ICE*	1.3	0.8	*TNP*	0.9	1.2						
Danofloxacin	*ICE*	0.19	0.00	*TNP*	0.0	0.14						
Enrofloxacin	*ICE*	0.27	0.00	*TNP*	0.0	0.22						
Florfenicol	*floR*	4.4 ^b^	0.3 ^a^	*ICE*	2.2	2.5	*TNP*	2.6	2.1			
Gentamicin	*ant(2″)-Ia*	NA^w^		*ICE*	17.2	10.3	*TNP*	13.3	14.2			
Neomycin	*aph(3′)-Ia*	42.8	40.3	*ICE*	51.2	31.9	*TNP*	34.9	48.2			
Oxytetracycline	*tet(H)*	6.0	6.7	*tet(R)*/ICE	13.9	0.0	*TNP*	4.6	8.1			
Penicillin	*bla_OXA-2_*	NA^x^		ICE	5.4	0.0	*TNP*	2.0	2.6			
Spectinomycin	*aadA25*	NA^x^		ICE	35.4	5.6	*TNP*	19.7	15.2			
Sullfadimethoxine	*sul*2	222.8	217.6	ICE	252.2	188.2	*TNP*	207.3	233.1			
Tilmicosin	*msr(E)* or *mph(E)*	78.6 ^b^	3.0 ^a^	*erm(42)*	43.1	38.6	ICE	51.2	31.9	*TNP*	10.7	11.4
Trimethroprim/ Sulfmethazole	*sul*2	0.01	0.00	ICE	0.01	0.00	*TNP*	0.00	0.01			
Tulathromycin	*msr(E)* or *mph(E)*	127.5	5.8	*erm(42)*	68.0	65.2	ICE	68.1	65.1	*TNP*	66.0	67.3
Tylosin	*msr(E)* or *mph(E)*	15.52	4.7	*erm(42)*	8.4	11.8	ICE	18.2 ^b^	2.0 ^a^	*TNP*	7.4	12.8

^a,b^ Means within a row with different superscripts differ (*p* < 0.05). ^z^ ICE, gene fragments for *integrase*, *hypothetical protein*, *multicopper oxidase* and *relaxase* all present at same frequency, *TNP* = transposase. ^y^*tet*(R) present as same frequency as 4/5 ICE core genes; ^x^ NA, no isolates positive for this gene; ^w^ NA, only one isolate positive for this gene(s).

**Table 8:** Effect of management ^z^ and year of study on minimum inhibitory concentrations (MICs) of antimicrobials for isolates of *Mycoplasma bovis* (*n* = 61). Least square means are reported for antimicrobials showing unimodal distributions (not shaded), and selected bimodal distributions (light grey shading). Arithmetic means are reported for other antimicrobials (dark grey shading). MICs < or > than the range of concentrations evaluated were estimated as the next lower or higher concentration commonly used on the Sensititre plate prior to statistical analyses.

Antimicrobial	CON	NAT	1	2	SEM
Chlortetracycline	4.2	3.3	3.8	3.6	0.9
Enrofloxacin	1.3	0.2	0.7	0.8	1.1
Florfenicol	4.4	2.9	3.7	3.6	0.9
Gamithromycin	239.0 ^b^	162.4 ^a^	245.9 ^b^	155.6 ^a^	12.3
Oxytetracycline	4.1	3.2	3.2	4.2	0.7
Tilmicosin	253.3	256.0	253.3	256.0	NA
Tidipirosin	251.1	256.0	251.1	256.0	NA
Tulathromycin	225.9 ^b^	141.3 ^a^	239.2 ^b^	128.0 ^a^	20.8
Tylosin	198.7	183.5	236.9	145.3	24.8

^a,b^ Means within a row with different superscripts differ (*p* < 0.05). ^z^ Management, CON = conventional, NAT = without antimicrobials; ^y^ NA, not applicable as arithmetic mean.

**Table 9:** Isolates of *M. haemolytica*, (MH), *P. multocida* (PM) and *H. somni* (HS) and antimicrobial resistance genes detected (+) or absent (-) by whole-genome sequence analyses (WGS). Genes with yellow shading were evaluated only by WGS. For genes with grey shading, PCR lacks concordance with WGS analyses.

Strain	*aph* (3′)-Ia	*aph* (6)-Id	*aph* (3″)-Ib	*sul2*	*floR*	*erm* (42)	*ant* (2″)-Ia	*aad* A31	*aad* A25	*bla*_OXA-2_	*bla*_ROB-1_	*mph (E)*	*msr (E)*	*tet (H)*	QRDR
MH-1	+	+	+	+	-	-	-	-	-	-	-	-	-	+	-
MH-2	+	+	+	+	-	-	-	-	-	-	-	-	-	+	-
MH-3	+	+	+	+	-	-	-	-	-	-	-	-	-	+	-
MH-4	+	+	+	+	-	-	-	-	-	-	-	-	-	+	-
MH-5	+	+	+	+	-	-	-	-	-	-	-	-	-	+	-
MH-6	+	+	+	+	-	-	-	-	-	-	-	-	-	+	-
MH-7	+	+	+	+	+	+	+	-	+	+	+	+	+	+	+ (GyrA^y^: S83F, D87N; ParC^y^: E89K)
MH-8	+	+	+	+	+	+	+	-	+	+	+	+	+	+	+ (GyrA: S83F, D87N; ParC: E89K)
MH-9	-	-	-	-	-	-	-	-	-	-	-	-	-	-	-
PM-1	+	+	+	+	-	-	-	+	-	-	-	-	-	+	-
PM-2	+	+	+	+	-	-	-	+	-	-	-	-	-	+	-
PM-3	+	+	+	+	-	-	-	+	-	-	-	-	-	+	-
PM-4	-	-	-	-	-	-	-	+	-	-	-	-	-	+	(GyrA: S88R)
PM-5	+	+	+	+	-	-	-	+	-	-	-	-	-	+	-
HS-1	-	+	+	+	-	-	-	-	-	-	-	+	+	+	-
HS-2	-	+	+	+	-	-	-	-	-	-	-	+	+	+	-
HS-3	-	+	+	+	-	-	-	-	-	-	-	+	+	+	-
HS-4	+	+	+	+	-	-	-	+	-	-	-	-	-	+	-
HS-5	-	+	+	+	-	-	-	-	-	-	-	+	+	+	-
HS-6	-	-	-	-	-	-	-	-	-	-	-	-	-	+	-
HS-7	+	+	+	+	+	-	-	-	-	-	-	-	-	+	-

^z^ Antimicrobial resistance genes, QRDR, quinolone resistance-determining region; ^y^ *GyrA* and *ParC*, QRDR amino acid changes.

## Data Availability

The draft whole-genome sequences of five PM, seven HS and nine MH isolates analyzed in this study have been deposited in GenBank under Bio Project PRJNA605035.

## Supplementary Materials

The following are available online at https://www.mdpi.com/2076-2607/8/2/288/s1. Table S1: Distribution of minimum inhibitory concentrations (MICS) of *Mycoplasma bovis* isolated from lung tissue (*n* = 61) of bovine respiratory disease mortalities. Table S2: Distribution of minimum inhibitory concentrations (MICS) of *Mannheimia haemolytica* (*n* = 104) isolated from lung tissue of bovine respiratory disease mortalities. Table S3: Distribution of minimum inhibitory concentrations (MICS) of *Pasteurella multocida* (*n* = 45) isolated from lung tissue of bovine respiratory disease mortalities. Table S4: Distribution of minimum inhibitory concentrations (MICS) of *Histophilus somni* (*n* = 23) isolated from lung tissue of bovine respiratory disease mortalities.
