# Supplementary material for: Antimicrobial Resistance in Members of the Bacterial Bovine Respiratory Disease Complex Isolated from Lung Tissue of Cattle Mortalities Managed with or without the Use of Antimicrobials
Source: Microorganisms. 2020 Feb 20;8(2):288. doi: 10.3390/microorganisms8020288 (PMC7074851; doi:10.3390/microorganisms8020288)
Supplement: Supplementary file 1 [file microorganisms-08-00288-s001.pdf]

Table S1. Distribution of minimum inhibitory concentrations (MICs) of *Mycoplasma bovis* isolated from lung tissue (n=61) of bovine respiratory disease mortalities. Dilution ranges tested are not shaded. Cells highlighted in yellow had growth at the highest concentration evaluated.

				MIC μg/mL											
Antimicrobial agent	Abbreviation	Manage[z]	S[y]	0.12	0.25	0.5	1	2	4	8	16	32	64	128	256
Chlortetracycline	CTET	CON	15					3	20	16					
		NAT	3						3	1					
Enrofloxacin	ENRO	CON	39		5			2	3	3	2				
		NAT	7												
Florfenicol	FFN	CON	2				4	12	22	13	1				
		NAT				1		2	4						
Gamithromycin	GAM	CON											1	6	47
		NAT									1		1	1	4
Oxytetracycline	OXY	CON	9					9	21	15					
		NAT	2				1		3	1					
Penicillin[x]	PEN	CON								54					
		NAT								7					
Tilimicosin	TIL	CON										1			53
		NAT													7
Tildipirosin	TIP	CON										1		53	
		NAT												7	
Tulathromycin	TUL	CON								3	3	1			47
		NAT									1	2			4
Tylosin tartarate	TYLT	CON	2						1	3	1	1	1	5	40
		NAT								1	1				5

[z]Manage, CON=conventional management with antimicrobials, NAT= without antimicrobials.

[y]S, susceptible to the lowest concentration evaluated.

[x]Used as a positive control.

Table S2. Distribution of minimum inhibitory concentrations (MICS) of *Mannheimia haemolytica* (n=104) isolated from lung tissue of bovine respiratory disease mortalities. Dilution ranges tested are not shaded. Cells highlighted in yellow had growth at the highest concentration evaluated.

			MIC μg/mL												
Antimicrobial	Abbreviation	Manage[z]	S[y]	0.12	0.25	0.5	1	2	4	8	16	32	64	128	256
Ampicillin	AMP	CON	47			5	1	2		1		42			
		NAT	6												
Ceftiofur	TIO	CON	72			16	7	1		1	1				
		NAT	4			2									
Clindamycin	CLI	CON								2	14	82			
		NAT									3	3			
Chlortetracycline	CTET	CON					34	7	16	39	2				
		NAT					3	1	1						
Danofloxacin	DANO	CON	47		4		4	43							
		NAT	6												
Enrofloxacin	ENRO	CON	50		1		4		43						
		NAT	6												
Florfenicol	FFN	CON	1			4	46	2	2	1	42				
		NAT					5	1							
Gentamicin	GEN	CON	46						7	1	44				
		NAT	4					1	1						
Neomycin	NEO	CON	4							31	1	1	61		
		NAT								4			2		
Oxytetracycline	OXY	CON	3				29	2		1	63				
		NAT	1				3				2				
Penicillin	PEN	CON	13		33	8	1				43				
		NAT	1		4	1									
Sulphadi methoxine	SDM	CON	1												97
		NAT	1												5
Spectinomycin	SPE	CON	1								3	47	2	45	
		NAT										6			
Trimethroprim/ Sulfamethoxazole	SXT	CON	28	70											
		NAT	4	2											
Tiamulin	TIA	CON	1							13	54	30	63		
		NAT									4	2			
Tilmicosin	TIL	CON	20							2	4	1	3	68	
		NAT	4								2				
Tulathromycin	TUL	CON	1					2	10	11	2	1	2	69	
		NAT							2	2	2				
Tylosin tartarate	TYLT	CON	3										95		
		NAT											6		

[z]Manage, CON=conventional management with antimicrobials, NAT =without antimicrobials.
[y]S, susceptible to the lowest concentration evaluated.

Table S3. Distribution of minimum inhibitory concentrations (MICS) of *Pasteurella multocida* (n=45) isolated from lung tissue of bovine respiratory disease mortalities. Dilution ranges tested are not shaded. Cells highlighted in yellow had growth at the highest concentration evaluated.

				MIC values μg/mL											
Antimicrobial agent	Abbreviation	Manage[z]	S[y]	0.12	0.25	0.5	1	2	4	8	16	32	64	128	> 256
Ampicillin	AMP	CON	3				2	7	16	3	1	9			
		NAT							2	1	1				
Ceftiofur	TIO	CON	2				6	10	8	3	12				
		NAT					2		1		1				
Clindamycin	CLI	CON										41			
		NAT									3	1			
Chlortetracycline	CTET	CON	2				6		32		1				
		NAT					4								
Danofloxacin	DANO	CON	22		6	3	6	4							
		NAT	4												
Enrofloxacin	ENRO	CON	31		2	7		1							
		NAT	4												
Florfenicol	FFN	CON	1			6	29	4			1				
		NAT				2		2							
Gentamicin	GEN	CON	3						25	12	1				
		NAT	3									1			
Neomycin	NEO	CON								7	11	1	22		
		NAT	2							2					
Oxytetracycline	OXY	CON	2				4	2			33				
		NAT	4												
Penicillin	PEN	CON	5				5	14	7	1	9				
		NAT					2	1		1					
Sulphadimethoxine	SDM	CON													41
		NAT													4
Spectinomycin	SPE	CON										7		34	
		NAT	2								2				
Trimethroprim/ Sulfamethoxazole	SXT	CON	9	32											
		NAT	2	2											
Tiamulin	TIA	CON								1	1	16	23		
		NAT									2	2			
Tilmicosin	TIL	CON								6	2	1		32	
		NAT	4												
Tulathromycin	TUL	CON	4					1		2	1		1	32	
		NAT	4												
Tylosin tartarate	TYLT	CON	3									4	34		
		NAT	2										2		

[z]Manage, CON=conventional management with antimicrobials, NAT=without antimicrobials.
[y]S, susceptible to the lowest concentration evaluated.

Table S4. Distribution of minimum inhibitory concentrations (MICS) of *Histophilus somni* (n=23) isolated from lung tissue of bovine respiratory disease mortalities. Dilutions tested are not shaded. Cells highlighted in yellow had growth at highest concentration evaluated. All mortalities received antimicrobials according to conventional feedlot management protocols.

MIC µg/mL

Antimicrobial agent	Abbreviation	S[z]	0.12	0.25	0.5	1	2	4	8	16	32	64	128	256	>256
Ampicillin	AMP	16				1	1	2	1	1	1				
Ceftiofur	TIO	18								5					
Clindamycin	CLI	4								10	6	1	2		
Chlortetracycline	CTET	7				10	5	1							
Danofloxacin	DANO	23													
Enrofloxacin	ENRO	2				6	12	2	1						
Florfenicol	FFN	8			1	1	4	8	1						
Gentamicin	GEN	22					1								
Neomycin	NEO	11							4	1		5	2		
Oxytetracycline	OXY	22						1							
Penicillin	PEN	22				1									
Sulphadimethoxine	SDM	16			1		4	2							
Spectinomycin	SPE	5							5	8	5				
Trimethroprim/ Sulfamethoxazole	SXT	5									5	13			
Tiamulin	TIA	3					4	6	2	1		7			
Tilmicosin	TIL	10								13					
Tulathromycin	TUL	2												21	
Tylosin tartarate	TYLT	0				1	3	4	5	7	2	1			

[z]S, susceptible to the lowest concentration evaluated.
